# A novel nomogram based on LODDS to predict the prognosis of epithelial ovarian cancer

**DOI:** 10.18632/oncotarget.14100

**Published:** 2016-12-22

**Authors:** Xue-Lian Xu, Hao Cheng, Meng-Si Tang, Hai-Liang Zhang, Rui-Yan Wu, Yan Yu, Xuan Li, Xiu-Min Wang, Jia Mai, Chen-Lu Yang, Lin Jiao, Zhi-Ling Li, Zhen-Mei Zhong, Rong Deng, Jun-Dong Li, Xiao-Feng Zhu

**Affiliations:** ^1^ State Key Laboratory of Oncology in South China, Collaborative Innovation Center for Cancer Medicine, Cancer Center, Sun Yat-sen University, Guangzhou 510060, China; ^2^ Department of Gynecological Oncology, Sun Yat-sen University Cancer Center, Guangzhou 510060, China; ^3^ The First Affiliated Hospital of Guangdong Pharmaceutical University, Guangzhou 510060, China

**Keywords:** LODDS, epithelial ovarian cancer, nomogram, prognosis, SEER

## Abstract

**BACKGROUND:**

To develop and validate a nomogram based on log of odds between the number of positive lymph node and the number of negative lymph node (LODDS) in predicting the overall survival (OS) and cancer specific survival (CSS) for epithelial ovarian cancer (EOC) patients.

**MATERIALS AND METHODS:**

A total of 10,692 post-operative EOC patients diagnosed between 2004 and 2013 were obtained from the Surveillance, Epidemiology, and End Results (SEER) database and randomly divided into training (n = 7,021) and validation (n = 3,671) cohorts. Multiple clinical pathological parameters were assessed and compared with outcomes. Parameters significantly correlating with outcomes were used to build a nomogram. Bootstrap validation was subsequently used to assess the predictive value of the model.

**RESULTS:**

In the training set, age at diagnosis, race, marital status, tumor location, stage, grade and LODDS were correlated significantly with outcome in both the univariate and multivariate analyses and were used to develop a nomogram. The nomogram demonstrated good accuracy in predicting OS and CSS, with a bootstrap-corrected concordance index of 0.757 (95% CI, 0.746-0.768) for OS and 0.770 (95% CI, 0.759-0.782) for CSS. Notably, in this population our model performed favorably compared to the currently utilized Federation of Gynecology and Obstetrics (FIGO) model, with concordance indices of 0.699 (95% CI, 0.688-0.710, P < 0.05) and 0.719 (95% CI, 0.709- 0.730, P < 0.05) for OS and CSS, respectively. Using our nomogram in the validation cohort, the C-indices were 0.757 (95% CI, 0.741-0.773, P < 0.05, compared to FIGO) for OS and 0.762 (95% CI, 0.746-0.779, P < 0.05, compared to FIGO) for CSS.

**CONCLUSIONS:**

LODDS works as an independent prognostic factor for predicting survival in patients with EOC regardless of the tumor stage. By incorporating LODDS, our nomogram may be superior to the currently utilized FIGO staging system in predicting OS and CSS among post-operative EOC patients.

## INTRODUCTION

Ovarian cancer is the fifth leading cause of cancer-related death in women around the world. It is estimated that 1,4240 deaths will be attributed to this disease according to the National Cancer Institute [1]. As early-stage ovarian cancer is typically asymptomatic, most patients are diagnosed at more advanced stages, with associated dismal prognosis.

Traditionally, lymph node status has been considered to be an important predictor of outcomes for patients with ovarian cancer [2, 3]. Presently, lymph node status is based on PLNs regardless of the number of resected lymph node (RLNs) in epithelial ovarian cancer (EOC) patient [4, 5]. Consequently, the actual lymph node status may not adequately be reflected by the number of positive lymph node (PLNs).

Recently, ratio of PLNs to RLNs (LNR) and log of odds between PLNs and the number of negative lymph node (LODDS) have emerged as alternative predictive factors for outcomes in breast [6, 7], gastric [8], pancreatic [9] and colorectal cancer [10, 11]. LNR is defined as the ratio of PLNs to RLNs. This parameter showed superiority to the lymph node status-based assessment system of N stage in several cancers, such as breast [6, 12], gastric [8], and colon cancer [13, 14]. LNR was also studied in ovarian cancer and found to be superior to both PLN and RLN number in predicting survival [15, 16].

LODDS is defined as the log of odds between PLNs and the number of negative node. While this parameter has been validated in predicting survival for breast cancer, its utility in epithelial ovarian cancer is unknown [7, 17, 18]. We hypothesized that LODDS might be an important predictor of outcomes in epithelial ovarian cancer and that a nomogram incorporating LODDS may be superior in this respect to the currently-utilized FIGO score.

## RESULTS

### Clinical characteristics

The clinicopathological characteristics of these patients are listed in Table 1. There were no significant differences between the training and validation cohorts. The median age at the time of diagnosis was 7 (49–66) years in both cohorts. Most patients are married (56.8%). The majority of patients were Stage III at the time of surgery (47.5%). The most frequent histologic type was serous (56.5%). A majority of patients (98.8%) did not receive radiotherapy. The mean values for RLNs, PLNs, LNR and LODDS were 14.37, 1.35, 0.13 and -0.99 respectively. Additionally, the median survival time was 38 (90–145) months.

**Table 1:** Clinicopathological characteristics of EOC patients in the training and validation cohort

Characteristic	All patients (*n* =10692)	Training cohort (*n* = 7021)	Validation cohort (*n* = 3671)	P
**Age at diagnosis, years**				0.63
Median (IQR)	57(49-66)	57(49-66)	57(49-66)	
**Year of diagnosis**				0.56
2004-2007	3936	2559	1377	
2007-2010	3261	2152	1109	
2010-2013	3495	2310	1185	
**Race**				0.17
White	9045	5906	3139	
Black	521	354	167	
Other*	1126	761	365	
**Marital status**				0.04
Married	6069	4042	2027	
Single	2085	1360	725	
Others#	2538	1619	919	
**FIGO Stage**				0.78
I	4172	2745	1427	
II	1445	937	508	
III	5075	3339	1736	
**Grade**				0.13
Well differentiate	1592	1051	541	
Moderately differentiate	2482	1581	901	
Poor differentiate	4482	2979	1503	
**Tumor location**				
One site	6738	4427	2311	0.87
Bilateral site	3859	2534	1325	
Paired site	95	60	35	
**Radiation**				
No	10565	6935	3630	0.70
Yes	127	86	41	
**Histology**				0.98
Serous	6037	3977	2060	
Mucinous	1022	669	353	
Endometrioid	2518	1642	876	
Clear cell	1054	692	362	
Undifferentiated	61	41	20	
**RLNs**				0.76
Mean (range)	14.37(1-90)	14.45(1-90)	14.23(1-90)	
**PLNs**				0.77
Mean (range)	1.35(0-90)	1.39(0-90)	1.26(0-57)	
**LNR**				0.72
Mean (range)	0.13(0.00-1.00)	0.13(0.00-1.00)	0.13(1.00-1.00)	
**LODDS**				0.86
Mean (range)	−0.99(-2.26-2.26)	−0.98(-2.26-2.26)	−0.99(-2.16-1.95)	

* Other including American Indian/AK Native, Asian/Pacific Islander.

# Other including Widowed, Divorced, Separated and unknown

Poor differentiate including Poor differentiate and undifferentiated

Abbreviations: RLNs, the number of lymph node examined; PLNs, the number of positive lymph node; LNR, positive lymph node ratio; LODDS, log of odds between the number of PLNs and number of negative nodes.

### Factors associated with OS

Age at diagnosis, race, marital status, year of diagnosis, tumor location, tumor stage, tumor grade, histology, RLNs, PLNs, LNR, LODDS and treatment with radiation therapy were evaluated. All variables were significantly correlated with OS in univariate survival analysis (P < 0.05). Independent prognostic factors associated with OS identified from the multivariate analysis were subsequently incorporated into the nomogram (Table 2).

**Table 2:** Characteristics and multivariate analysis in the training set

Variables	Univariate Analysis		Multivariate Analysis	
	HR (95% CI)	*P* value	HR (95% CI)	*P* value
Age at diagnosis, years	1.04(1.03-1.04)	<.001	1.028(1.024-1.032)	<.001
Race	0.90(0.84-0.97)	0.01		
Other*			Reference	
White			1.106(0.934-1.311)	0.242
Black			1.427(1.118-1.820)	0.004
Marital status	1.19(1.13-1.26)	<.001		
Others^#^			Reference	
Married			0.818(0.734-0.911)	<.001
Single			0.907(0.780-1.053)	0.200
Stage	2.46(2.30-2.62)	<.001		
III			Reference	
I			0.280(0.238-0.330)	<.001
II			0.469(0.395-0.557)	<.001
Grade	1.52(1.45-1.60)	<.001		
Poor differentiate			Reference	
Well differentiate			0.514(0.412-0.642)	.000
Mediate differentiate			0.845(0.744- 0.961)	.010
Tumor location	1.45(1.39-1.51)	<.001		
Paired site			Reference	
One site			0.897(0.591-1.361)	0.609
Bilateral site			1.154(0.762- 1.747)	0.500
Radiation	1.45(1.04-2.00)	0.03		
No				
Yes				
Histology	0.68(0.65-0.72)	<.001		
Undifferentiated			Reference	
Serous			0.686(0.421-1.118)	0.131
Mucinous			1.193(0.701 -2.031)	0.515
Endometrioid			0.613(0.3691-.019)	0.059
Clear cell			0.983(0.587-1.647)	0.949
RLNs	0.98(0.98-0.99)	<.001	0.995(0.991-0.999)	0.013
PLNs	1.04(1.04-1.05)			
LNR	4.67(4.15-5.27)			
LODDS	2.10(1.99-2.21)		1.359(1.269-1.454)	<0.001

* Other including American Indian/AK Native, Asian/Pacific Islander.

# Other including Widowed, Divorced, Separated and unknown.

Poor differentiate including Poor differentiate and undifferentiated.

Abbreviations: CI, confidence interval; HR, hazard ratio; RLNs, the number of lymph node examined; PLNs, the number of positive lymph node; LNR, positive lymph node ratio; LODDS, log of odds between the number of PLNs and number of negative nodes.

### Comparing the prognostic impact of RLNs, PLNs, LNR and LODDS

**Figure 1:**
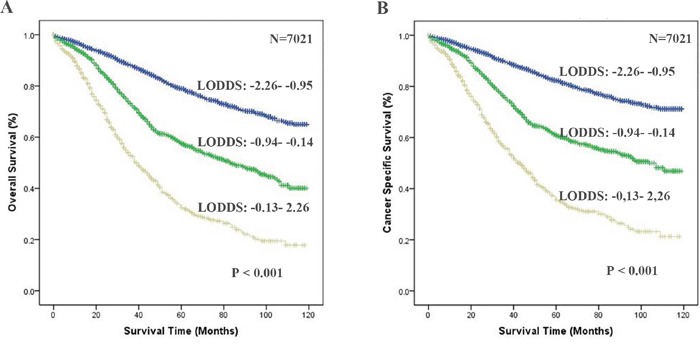
Comparison of the areas under curves of the number of RLNs, PLNs, LNR and LODDS to predict the impact of these factors on OS **A.** and CSS **B.** in the SEER training cohort. The green lines represent LODDS predicted survival, the blue lines represent the LNR predicted survival, the red line represent PLNs predicted survival and the black line represent RLNs predicted survival. Abbreviation: OS, overall survival; CSS, cancer specific survival; RLNs, the number of resected lymph node; PLNs, the number of positive lymph node; LNR, ratio of PLNs to RLNs; LODDS, log of odds between the number of positive lymph node (PLNs) and number of negative nodes.

**Figure 2:**
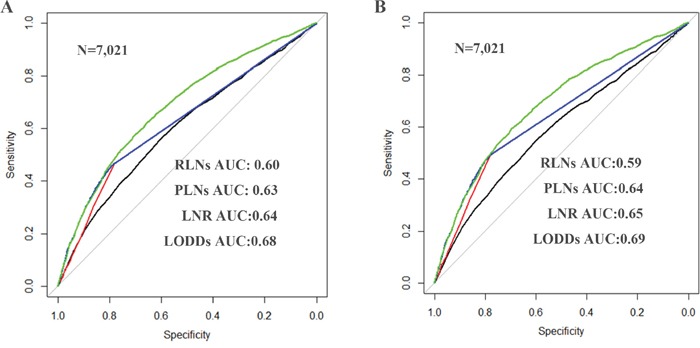
The overall survival (OS) and cancer specific survival (CSS) curves of epithelial ovarian cancer patients according to the cutoff points based on LODDS. OS **A.** and CSS **B.** are plotted using the Kaplan-Meier method and analyzed by the log-rank test (P<0.001). Abbreviation: OS, overall survival; CSS, cancer specific survival; LODDS, log of odds between the number of positive lymph node (PLNs) and number of negative nodes.

According to Figure 1, it is clearly that LODDS is a prognostic factor for EOC patients. The AUCs corresponding to inclusion of RLNs, PLNs, LNR and LODDS were 0.602, 0.625, 0.635 and 0.683 (respectively) for predicting OS and 0.590, 0.638, 0.646 and 0.686 (respectively) for predicting CSS (Figure 2), suggesting that LODDS may have superior discriminatory ability in predicting OS and CSS than the other parameters

### Nomogram

**Figure 3:**
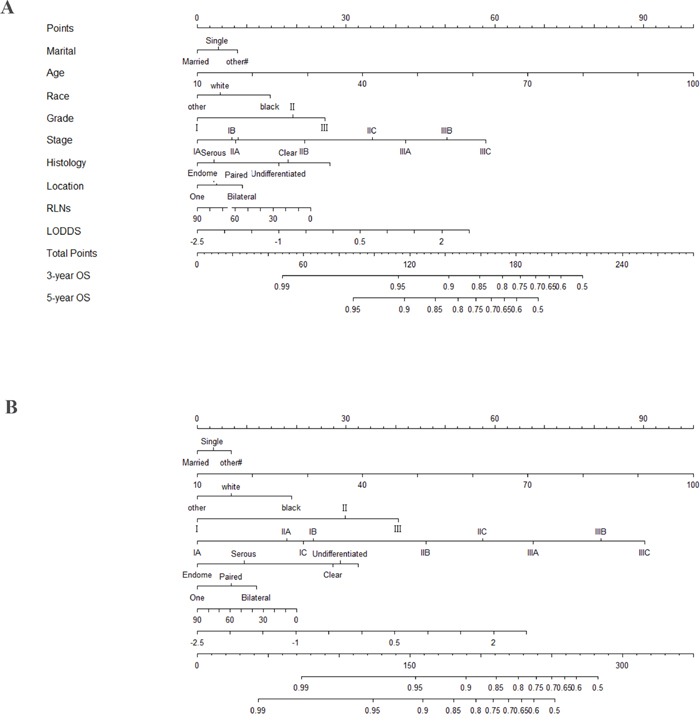
Nomogram for predicting 3- and 5-year **A.** cancer specific survival (CSS) and **B.** overall survival (OS) of epithelial ovarian cancer patients. To use of the nomogram, you should assign the points of each characteristic of the patient by drawing a vertical line from that variable to the point scale, sum all the points and draw a vertical line from the total points scale to the 3- and 5-year CSS or OS to obtain the probability of death. Abbreviation: OS, overall survival; CSS, cancer specific survival; LODDS, log of odds between the number of positive lymph node (PLNs) and number of negative nodes.

All of the 11 parameters were associated with overall survival analyzed in the univariate analysis in EOC patients (Table 2, P < 0.05). Nomograms for predicting 3- and 5 year OS and CSS were constructed based on the multivariate models by the backward method in the training cohort (Figure 3). By summing and locating the scores on the total score scale, the estimated probability of cancer-specific survival at 3- and 5-years could be determined. The bootstrap-corrected concordance indices were 0.75 (95% CI, 0.74-0.77) for predicting OS and 0.77 (95% CI, 0.76-0.78) for predicting CSS. Notably, the C-indices for FIGO stage were significantly lower: 0.699 (95% CI, 0.688-0.710, P < 0.05) for predicting OS and 0.719 (95% CI, 0.709- 0.730, P < 0.05) for predicting CSS.

### Validation for nomogram

**Figure 4:**
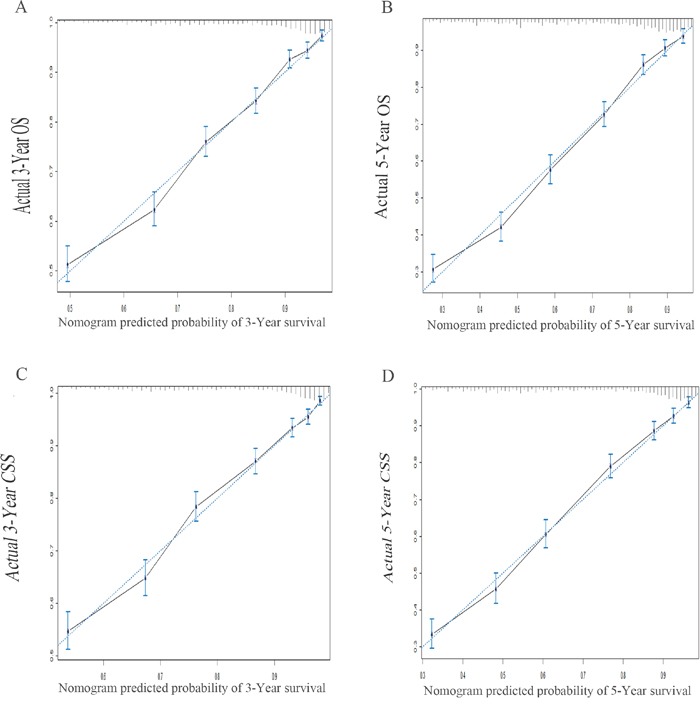
The calibration plots predicting overall survival **A-B.** and cancer specific survival **C-D.** at 3- and 5-year point in the training cohort. The dashed line represents a perfect match between the nomogram predicted probability (x-axis) and the actual probability calculated by Kaplan-Meier analysis (y-axis). Abbreviation: OS, overall survival; CSS, cancer specific survival.

**Figure 5:**
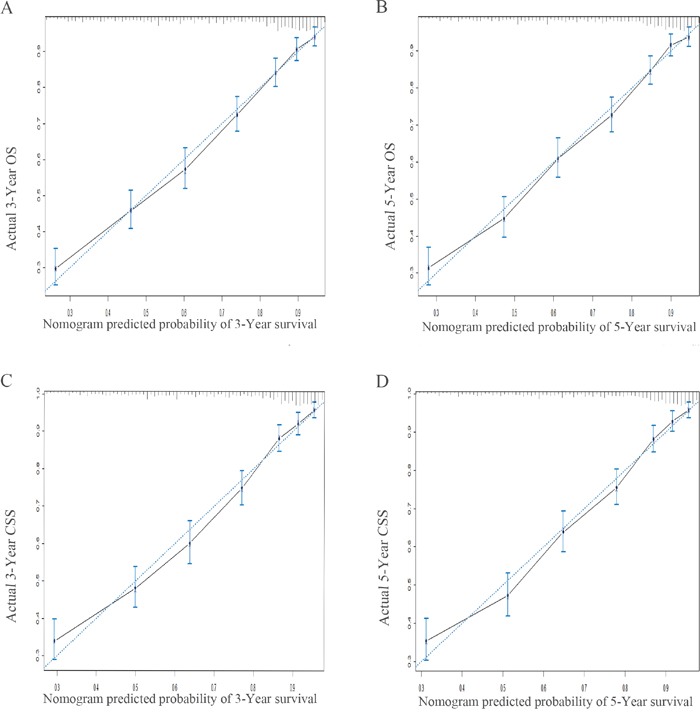
The calibration plots predicting overall survival A-B. and cancer specific survival C-D. at 3- and 5-year point in the validation cohort. The dashed line represents a perfect match between the nomogram predicted probability (x-axis) and the actual probability calculated by Kaplan-Meier analysis (y-axis). Abbreviation: OS, overall survival; CSS, cancer specific survival.

**Figure 6:**
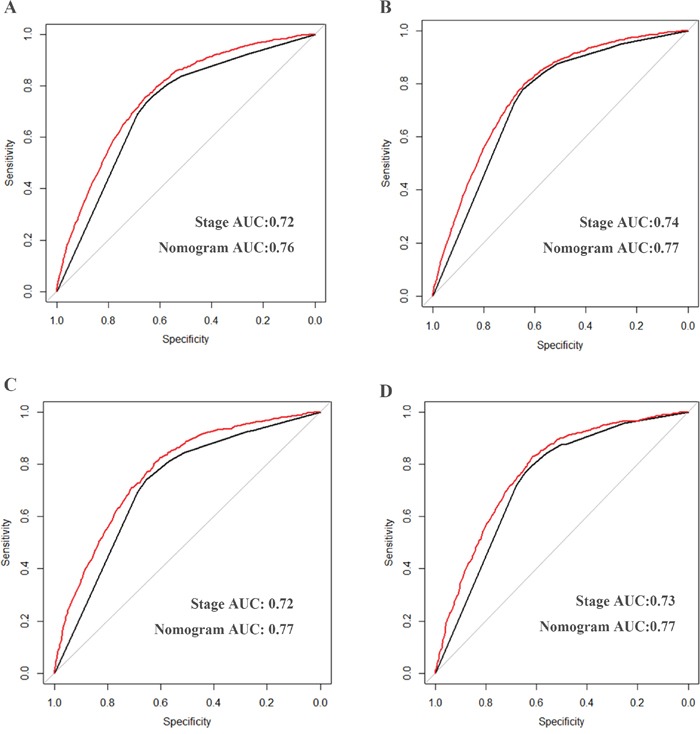
Comparison of the areas under curves of nomogram and Stage to prediction of OS **A, C.** and CSS **B, D.** in the training (A, B) and validation cohort (C, D). The red lines represent nomogram predicted survival and the black lines represent the traditional stage predicted survival. Abbreviation: OS, overall survival; CSS, cancer specific survival.

Validation of the nomogram was performed by 1000 bootstrap. In the training cohort, the Harrell's C-indices for the nomogram to predict OS and CSS were 0.757 (95% CI, 0.746-0.768) and 0.770 (95% CI, 0.759-0.782), respectively. Similarly, in the validation cohort, the C-indices to predict OS and CSS were 0.757 (95% CI, 0.741-0.773) and 0.762 (95% CI, 0.746-0.779). This finding implied that this model was reasonably accurate. The calibration plots demonstrated excellent correlation between predicted and observed values of OS and CSS in both training and validation cohorts (Figure 4 & Figure 5). Moreover, when comparing the c-index for OS and CSS based on either nomogram or FIGO stage, our nomogram showed better predictive probability than FIGO stage (Figure 6).

## DISCUSSION

In this study, a novel nomogram based on LODDS was developed and validated to predict post-operative outcomes in epithelial ovarian cancer. In training and validation cohorts that were randomly extracted from the SEER database, this nomogram appears to predict OS and CSS more accurately than the currently utilized FIGO staging system.

A nomogram is a convenient tool for quantifying risk by incorporating and illustrating the relative importance of various prognostic factors and has been widely used in clinical oncology [19]. Several nomograms have been constructed to date to predict outcomes in ovarian cancer patients [20–23]. To our knowledge, however, a nomogram based on LODDS for predicting outcomes in epithelial ovarian cancer patients has not been described.

It is well known that lymph node status can be used for post-operative risk stratification in epithelial ovarian cancer patients. The currently-utilized FIGO staging system uses PLNs in this calculus [4]. PLNs, however, remain a function of RLNs, and may not adequately reflect disease status in all situations [24]. For this reason, it is intuitive that RLNs and the number of negative lymph nodes should be simultaneously taken into consideration when formulating an adjuvant treatment plan. LODDS is an intuitive indicator that is reflective of both interrogated lymph nodes and the number of negative lymph nodes, and its use is supported by available data in other malignancies [7, 9–11]. For the first time in the epithelial ovarian cancer population, we evaluated ROC curves based on RLNs, PLNs, LNR and LODDS, and found that LODDS has the highest AUC with superior sensitivity and specificity compared to other factors.

We subsequently constructed a nomogram where patients were randomly assigned to either training or validation cohorts to avoid selection bias.

To date, several nomograms have been constructed for predicting outcomes in EOC patients that possess superior predictive ability compared to the widely-utilized FIGO staging system. In 2012, Barlin and colleagues evaluated for parameters predicting disease-specific survival after surgery based on outcomes in 478 EOC patients [25]. Lee et al. evaluated parameters predicting survival in patients initially responsive to a platinum-based regimen subsequently demonstrating recurrent disease in 2013 [26]. More recent studies evaluated survival based on surgery type [22, 23].

However, all of these nomograms were based on very limited cases. Thus, it is unclear whether it can be generally applied. Actually, sufficient data included is useful to improve the accuracy of nomogram. Compared with previous nomogram, our nomogram was developed and validated based both on a larger population on seer database which included several departments and racial might be universally applied.

The C-indexes for the nomograms to predict OS and CSS were 0.757, 0.770 respectively in the training cohort and 0.74, 0.76 respectively in the validation cohort which higher than FIGO stage. Therefore, this nomogram model has less bias and better accuracy when applied in practical work.

Improving the accuracy of the survival estimation is exceedingly important for clinical decision. Several advantages can be obtained by using nomogram. Firstly, as our nomogram performed better than FIGO stage on prediction of individual survival, it would be useful for designing postoperative treatment and the probability of excessive treatment would be reduced. Secondly, because our nomogram could calculate 3–year and 5–year survival rate individually, a more reasonable follow–up schedule could be obtained. Thirdly, individual consultant is in need of nomogram. The survival of EOC could not be accurately predicted by FIGO stage. In comparison, our nomogram can provide individualized estimation for EOC cancer patients.

Although the nomogram model demonstrated good accuracy for predicting OS and CSS, there are several limitations to the data which must be considered. Firstly, this study based on retrospective data which has inevitable inherent bias. Secondly, some prognostic factors like chemotherapy data and tumor marker like CA-125 which were not available. Finally, external validation should be carried out. In summary, our study demonstrated that LODDS was independently associated with the prognosis of EOC patients. We developed and validated a nomogram based on LODDS to estimate 3- and 5-year OS and CSS among EOC which could successfully stratify patients according to their OS and CSS. This clinically useful tool uses available clinicopathologic factors to estimate the probability of OS and CSS, which can further contribute to the individualized clinical decision.

## MATERIALS AND METHODS

Data were collected from the SEER program of the National Cancer Institute consisting of 18 population based cancer registries, which covers approximately 28% of the US population (http://seer.cancer.gov/). All pathologically-confirmed and surgically-treated EOC patients from the SEER database between 2004 and 2013 were included. Patients with unknown or incomplete lymph node status were excluded. A total of 10,692 patients with histologically confirmed EOC were obtained. These cases were then randomized to two groups (training cohort, N=7,021 and validation cohort, N=3,671). Patients in American Indian/Alaskan Native and Asian/Pacific Islanders were recorded as “other” under race. Widowed, Divorced, Separated and unknown were recorded as “other” under marital status. Poor differentiate and undifferentiated were classified as the poor differentiate group.

Our study was approved by Ethical Committee of Sun Yat-Sen University Cancer Center. Cancer is a reportable disease in every state in the US, informed patient consent is not required for the data released by the SEER database.

### Statistical analysis

### Construction of the nomogram

Eligible patients were randomly divided into training (n = 7,021) and validation cohorts (n= 3,671) to establish and validate the nomogram. The follow-up time was defined as the time between the surgery and the last follow-up time. OS was defined as the time between the surgery and cancer-specific death or the last follow-up time. CSS was defined as the time between the surgery and cancer-specific death. The Kaplan-Meier method and Cox regression analysis were used to determine factors associated with survival. Significant factors were used to construct the nomogram for OS and CSS.

### Validation of the nomogram

1000 bootstrap were performed for validation. Calibration diagrams were created with the marginal estimate versus model average predictive probability. The predictions should fall on a 45-degree diagonal line in a perfectly calibrated model. An index of probability of concordance (C-index) between predicted probability and actual outcome was calculated to evaluate the predicting ability and discrimination of the model [27]. The value of the C-index ranges from 0.5 to 1.0, with 0.5 indicating random chance and 1.0 indicating a perfectly corrected discrimination.

Statistical analysis was performed using SPSS 19.0 (SPSS, Chicago, IL, USA) and R version 3.3.0 software (Institute for Statistics and Mathematics, Vienna, Austria;www.r-project.org). The “rms” R library by Harrell (cran.r-project.org/web/packages/rms) was used to construct survival models [28]. P value less than 0.05 were considered statistically significant.
